# Alterations of NMR-Based Lipoprotein Profile Distinguish Unstable Angina Patients with Different Severity of Coronary Lesions

**DOI:** 10.3390/metabo13020273

**Published:** 2023-02-14

**Authors:** Yongxin Ye, Jiahua Fan, Zhiteng Chen, Xiuwen Li, Maoxiong Wu, Wenhao Liu, Shiyi Zhou, Morten Arendt Rasmussen, Søren Balling Engelsen, Yangxin Chen, Bekzod Khakimov, Min Xia

**Affiliations:** 1 Department of Nutrition, School of Public Health, Sun Yat-sen University (Northern Campus), Guangzhou 510080, China; 2 Guangdong Provincial Key Laboratory of Food, Nutrition and Health, Guangzhou 510080, China; 3 Department of Cardiology, Sun Yat-sen Memorial Hospital, Sun Yat-sen University, Guangzhou 510120, China; 4 Department of Medical Statistics and Epidemiology, School of Public Health, Sun Yat-sen University, Guangzhou 510080, China; 5 Department of Food Science, University of Copenhagen, 1958 Frederiksberg C, Denmark; 6 COPSAC—Copenhagen Prospective Studies on Asthma in Childhood, Herlev and Gentofte Hospital, University of Copenhagen, 2100 Copenhagen, Denmark

**Keywords:** NMR, lipoproteins, cardiovascular diseases, unstable angina, Gensini score, coronary lesions

## Abstract

Non-invasive detection of unstable angina (UA) patients with different severity of coronary lesions remains challenging. This study aimed to identify plasma lipoproteins (LPs) that can be used as potential biomarkers for assessing the severity of coronary lesions, determined by the Gensini score (GS), in UA patients. We collected blood plasma from 67 inpatients with angiographically normal coronary arteries (NCA) and 230 UA patients, 155 of them with lowGS (GS ≤ 25.4) and 75 with highGS (GS > 25.4), and analyzed it using proton nuclear magnetic resonance spectroscopy to quantify 112 lipoprotein variables. In a logistic regression model adjusted for four well-known risk factors (age, sex, body mass index and use of lipid-lowering drugs), we tested the association between each lipoprotein and the risk of UA. Combined with the result of LASSO and PLS-DA models, ten of them were identified as important LPs. The discrimination with the addition of selected LPs was evaluated. Compared with the basic logistic model that includes four risk factors, the addition of these ten LPs concentrations did not significantly improve UA versus NCA discrimination. However, thirty-two selected LPs showed notable discrimination power in logistic regression modeling distinguishing highGS UA patients from NCA with a 14.9% increase of the area under the receiver operating characteristics curve. Among these LPs, plasma from highGS patients was enriched with LDL and VLDL subfractions, but lacked HDL subfractions. In summary, we conclude that blood plasma lipoproteins can be used as biomarkers to distinguish UA patients with severe coronary lesions from NCA patients.

## 1. Introduction

Unstable angina (UA), a high-risk clinical manifestation of acute coronary syndromes (ACS), is a main cause of emergency medical services and hospital care. Unstable angina is characterized by severe, prolonged angina pain at rest or with minimal exertion, without detectable myocardial necrosis. Although UA patients have lower early mortality and one-year incidence rates of myocardial infarction (MI) than non-ST elevation myocardial infarction (NSTEMI) or ST-elevation (STEMI) patients, half of them develop recurrent coronary ischemia requiring revascularization, leading to the development of severe morbidity and a higher health burden [1]. The identification of UA patients with severe coronary lesions who are at a high risk of worse outcomes during hospitalization may improve their prognosis by implementing earlier and more precise treatments (e.g., by targeting high-risk patients for more aggressive therapy) [2].

Observational studies show a robust association between lipoprotein cholesterol levels, such as high-density lipoprotein cholesterol (HDL-C), and low-density lipoprotein cholesterol (LDL-C), and the risk factors of cardiovascular disease (CVD) in the healthy population and in secondary prevention [3,4]. These lipoproteins (LPs) participate in atherogenesis and form the cornerstone of lipoprotein-based risk stratification and lipid-lowering treatment in CVD [2]. However, HDL cholesterol-raising or LDL cholesterol-lowering therapeutics have failed to improve patients’ prognosis in multiple clinical trials [5,6]. Additionally, HDL-C and LDL-C have not consistently predicted the risk of major adverse cardiovascular outcomes. Recent large cohort studies showed that there are U-shaped associations between CVD risk and HDL-C or LDL-C due to residual atherosclerotic risk in patients with ACS [7,8]. Therefore, the traditional lipoprotein parameters are not enough to fully understand the mechanisms of the development of CVD. Recent observation studies [4,9] and experimental studies [10,11] reported that lipoprotein characteristics (e.g., sizes, densities, concentrations, core lipid composition, specific apolipoproteins and functions) may have different associations with future cardiovascular events, indicating lipoprotein subfractions and different compositions play various roles in the development of CVD. Thus, investigating different lipoprotein particles (LPs) and the corresponding components, may provide more information and be potential contributors to CVD risk stratification and therapy.

There are several methods for the measurement of different LP fractions, such as ultracentrifugation, high-performance liquid chromatography (HPLC) and one-dimensional proton nuclear magnetic resonance (^1^H NMR) spectroscopy. Among them, ^1^H NMR spectroscopy provides sufficient physicochemical information to identify LPs, and enables faster, inexpensive and reproducible quantification [12]. Recently, it has been successfully used in the evaluation of the relationship between LPs and the risk of CVD [13], cardiovascular events and mortality [14] and hypertriglyceridemia [15]. Regarding UA, a few studies related serum metabolomics profiles and UA [16,17], but they mainly focused on the main fraction of lipoproteins (VLDL, LDL, HDL) and their relative concentrations. Some other studies investigated the lipoprotein particles with cardiovascular diseases, but were not specific to UA [3,18,19], or they were more related to UA metabolomics [20]. The correlation between lipoprotein particles and UA patients with different plaque burdens remains unclear. Therefore, further investigations of the association between LPs’ composition and the risk of CVD may provide a more comprehensive understanding of the pathology of CVD progression and the residual cardiovascular risk [21], which will contribute to improving the identification of the degree of coronary lesions in patients with unstable angina at an early stage without using unnecessary invasive therapies and resources.

In this study, we used ^1^H NMR spectroscopy to measure blood plasma LPs of 230 unstable angina patients. The level of severity of coronary lesions was represented by the Gensini score (GS), which is a comprehensive indicator for describing the plaque burden and the severity of coronary lesions [22,23], based on their coronary angiography results. Subsequently, patients were classified based on the Gensini score into lowGS (n = 155, GS ≤ 25.4) and highGS (n = 75, GS > 25.4). The inpatients with angiographically normal coronary arteries (NCA, n = 67, GS = 0) were defined as a disease control group, in order to identify unique LP signatures for patients with UA with different severity levels of coronary lesions.

## 2. Materials and Methods

### 2.1. Study Population

The population of the present study was a subset of patients in an ongoing hospital-based cohort, the Guangdong Cardiovascular Disease Cohort. Briefly, the inpatients in the Department of Cardiology of Sun Yat-sen Memorial Hospital (Guangzhou, China) were recruited from 2017 to 2019 [24]. Anthropometric variables, medical records, medication history of lipid-lowering drugs, questionnaires and fasting blood samples of each participant were collected at the baseline survey, as previously described [24]. According to the 2015 ESC guideline [25], the inclusion criteria for UA patients was a history of angina pectoris (within one month), severe, prolonged anginal pain with minimal exertion or at rest and no increase of circulating troponin. The NCA patients were identified as inpatients with no lesions in their coronary arteries but who were suffering from unexplained chest pain as the control group. In order to control potential confounding factors that might affect the synthesis and metabolism of lipoprotein [8,26,27], the exclusion criteria included myocardial infarction, cirrhosis, malignant tumors, a recent surgical procedure, pulmonary embolism, autoimmune disorders, aortic dissection, severe infectious diseases, left ventricular ejection fraction < 20%, alanine aminotransferase level (ALT) > 135 U/L and creatinine > 3.0 mg/dL.

**Figure 1:**
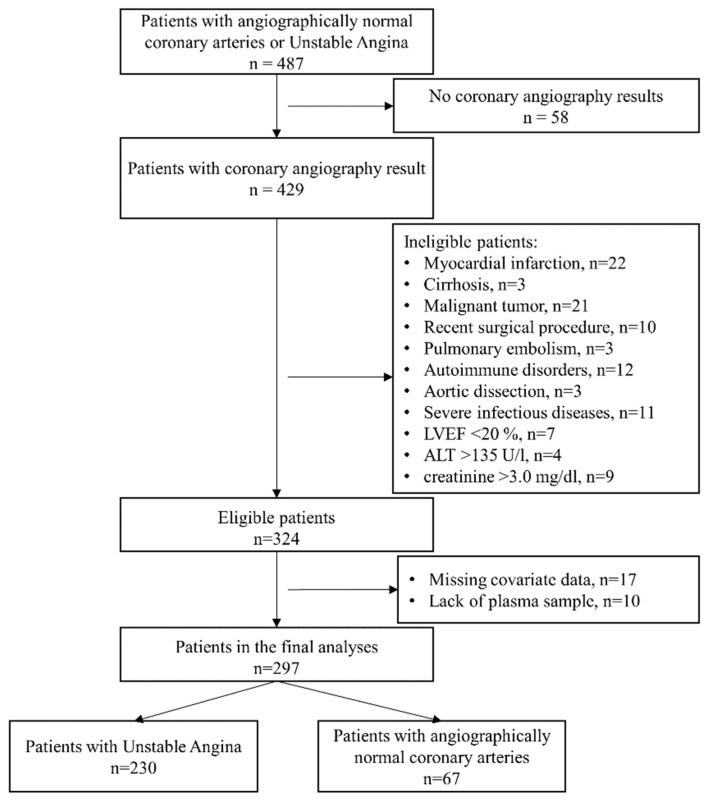
Flowchart of patient enrollment for the final study from the Guangdong Cardiovascular Disease Cohort. ALT, alanine transaminase; LVEF, left ventricular ejection fraction.

The present study included 487 patients. Of those, 190 patients were subsequently excluded due to non-coronary angiography results, above-mentioned comorbidities, missing covariate data and lack of enough plasma sample for NMR measurement, leaving 297 patients with UA (n = 230) and NCA (n = 67) eligible for the final analysis. The selection of patients for this analysis is detailed in Figure 1. Patients’ angiographic data were transformed into a Gensini score [23]. To investigate the associations between lipoprotein particles and the severity of coronary lesions in UA, the UA patients were divided into a lowGS group (n = 155, GS ≤ 25.4) and a highGS group (n = 75, GS > 25.4) based on the mean Gensini score, while NCA patients (n = 67, GS = 0) were used as a control group. The cohort and the analysis were conducted according to the Declaration of Helsinki and approved by the Sun Yat-sen University ethics committee. All patients signed the statement of informed written consent.

### 2.2. Clinical Measurements

The serum concentration of ALT, aspartate aminotransferase (AST), fasting glucose, fibrinogen, creatinine, high-sensitivity C-reactive protein (hs-CRP), lipid and apolipoprotein levels, including total cholesterol (TC), total triglyceride (TG), HDL-C, LDL-C, apolipoprotein A1 (Apo-A1), apolipoprotein B (Apo-B) and apolipoprotein E (Apo-E), were measured by an autoanalyzer (Beckman Coulter chemistry analyzer AU5800, Beckman Coulter Co., Ltd., Tokyo, Japan). Glycated hemoglobin (HbA1c) was determined using high-performance liquid chromatography (Variant II; Bio-Rad Laboratories, Hercules, CA, USA). High-sensitivity Troponin T (hs-TnT) was measured using standard clinical chemistry assays by an automated system (Roche Cobas e601, Hoffmann-La Roche Ltd., Basel, Switzerland).

### 2.3. Measurement of ^1^H NMR Spectra of Plasma

Plasma was centrifuged (3000 rpm, 4 °C, 15 min) after collection and stored at −80 °C until analysis. A 0.5 mL aliquot of each plasma sample was transferred on dry ice to the NMR laboratory (Tianjin, China). Of the 297 individuals, an extra quality control pooled plasma sample was prepared from 40 randomly selected individuals. As described previously, ^1^H NMR spectra of plasma samples were measured by Bruker Avance III 600 MHz NMR spectrometer (Bruker Biospin Gmbh, Rheinstetten, Germany) [28]. NMR spectra were obtained by NOESY-presat pulse sequences (noesygppr1d) from Bruker’s library. A total of 112 lipoprotein variables were quantified from the 1D NOESY 1H NMR spectra using the Bruker IVDr LIpoprotein Subclass Analysis (B.I.-LISA) prediction model [29], either as absolute concentrations or as ratios. This model determines triglycerides (*tg*), cholesterol (*chol*), free cholesterol (*fchol*) and phospholipids (*phol*), apolipoprotein A1, A2, B and particle numbers for different LPs.

Lipoprotein particles include four main fractions and their corresponding subfractions, which are intermediate density lipoprotein (IDL), very low-density lipoprotein (VLDL) and the five subfractions (VLDL-1 to VLDL-5), low-density lipoprotein (LDL) and six subfractions (LDL-1 to LDL-6), and high-density lipoprotein (HDL) and four subfractions (HDL-1 to HDL-4) [29]. All samples were randomly analyzed.

### 2.4. Statistical Analysis

Continuous variables are summarized with the means and standard deviation (SD) or medians with interquartile range (IQR). Independent Student’s t-test or Wilcoxon rank sum test was applied for the comparisons of continuous variables, and the chi-square test for categorical variables. Pearson’s correlation coefficients were applied for the agreement of lipoprotein variables measured by both ^1^H NMR and clinical chemistry.

ANOVA-simultaneous component analysis (ASCA) [30] was performed to evaluate the significance of the main effect of risk factors. Sex, age, BMI and the use of lipid-lowering drugs that had significant effects (*p* < 0.05) on LP data based on the ASCA result (Figure S1) were considered confounders and were adjusted in the following logistic regression analysis. This analysis was performed to assess the relationships of lipoprotein variables with the risk of UA and its subgroups, adjusted by the above four risk factors. The Benjamini and Hochberg false-discovery rate [31] adjusted *p*-value (FDR-p) of <0.1 was considered as significant. For each lipoprotein variable, the adjusted odds ratios (OR) with 95% confidence intervals (CI) were expressed per SD increment.

To allow for the potential confounding brought by correlated lipoprotein variables, two methods were conducted for variable selection. First, we applied multiple logistic regression with least absolute shrinkage and selection operator (LASSO) for variable selection [32] to first select the lipoprotein variables that contributed most to the associations with UA progression. LASSO is a shrinkage method that can select a set of more related and explainable variables from large and multi-collinear data and obtain an optimal model. Twenty-fold cross-validation was conducted using LASSO to yield the most predictive variables selected by the minimum (λ min). By taking advantage of LASSO, we then explored a panel of lipoprotein variables that are the most effective in distinguishing between the NCA group and the UA group, NCA and UA subgroups and lowGS and highGS groups, which could reflect UA progression.

Secondly, partial least squares discriminant analysis (PLS-DA) [33] was conducted to select significant lipoprotein variables discriminating patients in the different UA groups from the NCA group. Model performance was evaluated by the double cross-validation procedure [34]. PLS-DA can account for multi-collinearity among lipoprotein variables which is a problem that LASSO cannot handle. Lipoprotein data were normalized using mean centering and unit variance scaling before building the PLS-DA model. A ten-fold cross-validation was then conducted to find the best number of latent variables for PLS-DA models during the model-building process. A tenth of the samples were split out of every round and the remaining portion was used for the model validation. To identify the variables that discriminated the patients from different disease groups, the variable importance in the projection (VIP) scores from PLS-DA models were calculated.

To identify the key LP variables for differentiating the UA patients (UA, lowGS and high GS) from the NCA group, the distinguishing LP variables were selected under the following conditions: (1) had significant OR in logistic regression and VIP > 1 in PLS-DA, or (2) had significant OR in logistic regression and selected by LASSO model, or (3) had VIP > 1 in PLS-DA and selected by LASSO model. The selected LP variables were combined as a panel for the discrimination of two groups (UA versus NCA, lowGS versus NCA and highGS versus NCA).

Further, the selected LP variables were used in multivariate logistic regression analysis to test their impact on differentiating UA or UA subgroups from NCA (UA versus NCA, lowGS versus NCA and highGS versus NCA). For each comparison, three logistic regression models were built. Firstly, sex, age, BMI and the use of lipid-lowering drugs were investigated in model 1. Then the combined LP variables, selected from combinations of logistics regression, LASSO, and PLS-DA analyses performed in two groups (UA versus NCA, lowGS versus NCA and highGS versus NCA), were tested in model 2. Model 3 was adjusted for the combined selected LP variables and the four risk factors used in model 1. The area under the receiver operator characteristic curve (AUCROC) was calculated for assessing the accuracy of the added value of the selected lipoprotein variables for UA progression. Receiver operating characteristic (ROC) curves of different models were built and their corresponding AUCROC were tested using the Delong test.

Sensitivity analysis was applied to the patients without lipid-lowering treatment at baseline (n = 223) using logistic regression analysis adjusted by sex, age and BMI.

Partial Pearson’s correlation coefficient analyses were conducted to assess the relationship of each LP with the Gensini score and CVD risk biomarkers, including glucose, HbA1c, creatinine, hs-CRP, fibrinogen and hs-TnT, using the same adjustments as mentioned previously.

PLS-DA analyses were performed in the PLS Toolbox^TM^ (version 8.6.2—Eigenvector Research Inc., Manson, WA, USA) based on MATLAB R2016b. The LASSO regression [35] and Delong test were applied in R software (version 4.1.0). Logistic regression analyses and other analysis were conducted in MATLAB R2016b (MathWorks, Natick, MA, USA) using customized in-house scripts.

## 3. Results

### 3.1. Baseline Characteristics

Table 1 summarizes the baseline clinical characteristics of the patients. UA patients had higher serum concentrations of HbA1c, glucose, creatinine and hs-TnT than NCA patients. In contrast, lower levels of Apo-A1 and HDL-C were observed in UA groups. There was a progressive increase in stenosis vessels (*p* < 0.05) in lowGS to highGS groups.

The value of six LPs, including total triglycerides (TG), total cholesterol (TC), HDL-C, LDL-C, Apo-A1 and Apo-B, measured by clinical assays and 1H NMR showed good agreement (r = 0.80–0.93, Table S1). The absolute levels and IQR of lipoprotein variables in different groups are shown in Table S2.

### 3.2. Lipoprotein Particles Discriminating Unstable Angina from Angiographically Normal Coronary Arteries Patients

**Figure 2:**
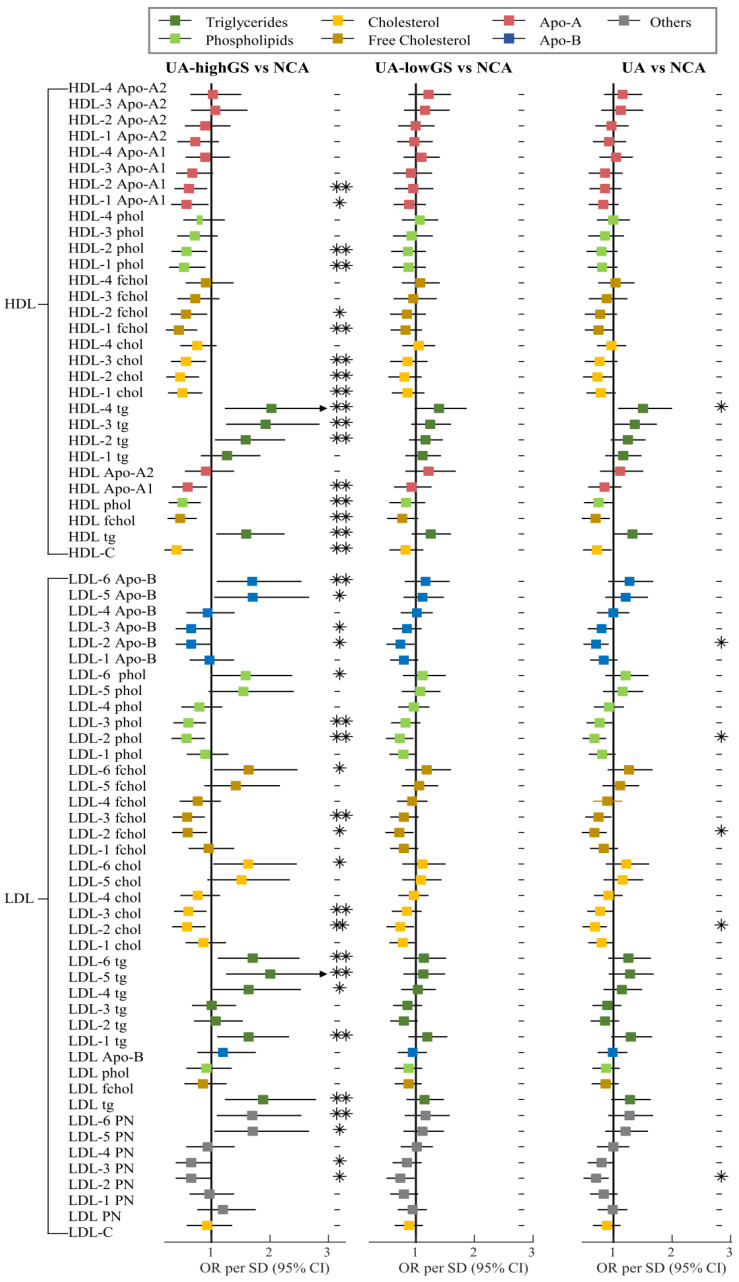

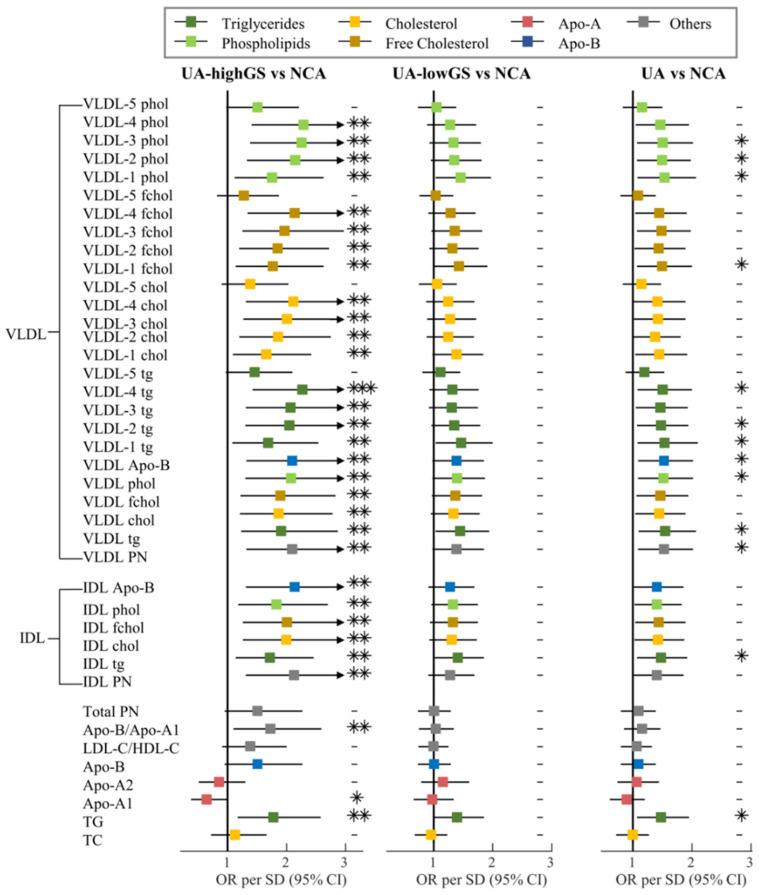
Associations of 112 Lipoprotein Variables with Unstable Angina (UA) and UA with Different Gensini Scores. Data are adjusted odds ratios (OR, blue dots) with 95% confidence intervals (black lines) per 1-SD (standard deviation) higher levels of lipoprotein variables, which were obtained by logistic regression models adjusting for sex, age, body mass index and the use of lipid-lowering drugs. Significance (Sig.): ** FDR-p < 0.05, * FDR-p < 0.1, FDR-p > 0.1 (*p* value). Apo-A1, apolipoprotein A1; Apo-A2, apolipoprotein A2; Apo-B, apolipoprotein B; chol, cholesterol; fchol, free cholesterol; HDL-C, high-density lipoprotein cholesterol; highGS, unstable angina patients with high Gensini score; IDL-C, intermediate-density lipoprotein cholesterol; LDL-C, low-density lipoprotein cholesterol; lowGS, unstable angina patients with low Gensini score; phol, phospholipids; PN, particle number; TC, total cholesterol; TG, total triglycerides; tg, triglycerides; VLDL-C, very low-density lipoprotein cholesterol.

Our results showed that 19 of 112 lipoprotein variables had significant associations with the risk of UA (FDR-p < 0.1) in logistic regression analysis, indicating that patients with lower levels of 5 LP variables (LDL-2 particle numbers, *chol*, *fchol*, *phol* and Apo-B) and higher levels of another 14 LP variables were more likely to develop UA (Figure 2).

**Figure 3:**
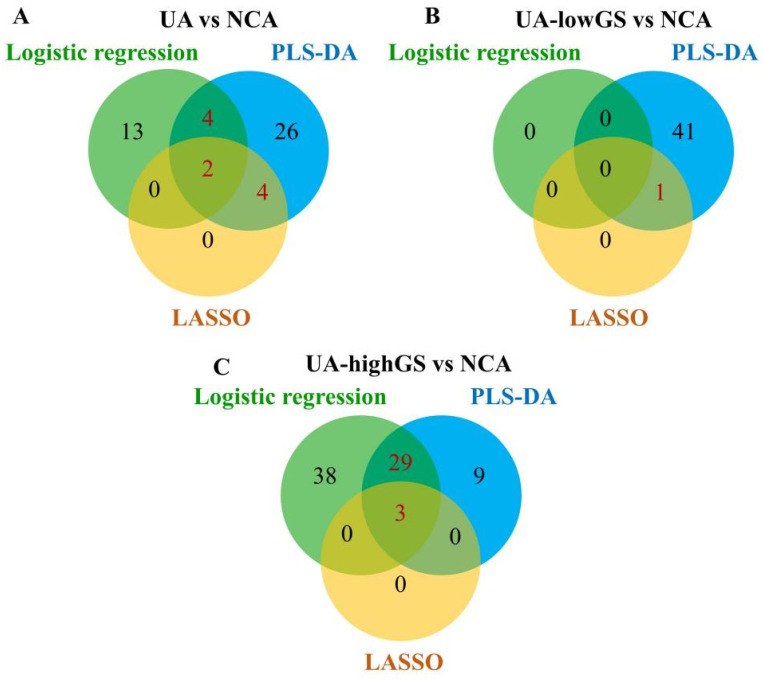
Venn diagram of the number of unique and shared lipoprotein variables selected by adjusted logistic regression, partial least squares discriminant analysis (PLS-DA), and least absolute shrinkage and selection operator (LASSO) for discriminating (**A**) unstable angina patients from NCA group, and also for discriminating (**B**) the UA patients with low Gensini score (GS) and (**C**) the UA patients with high GS from NCA group separately. highGS, unstable angina patients with Gensini score ≥ 25.4; lowGS, unstable angina patients with Gensini score < 25.4; NCA, normal coronary arteries; UA, unstable angina.

A panel of 36 lipoprotein variables was selected as the strongest classifiers from the PLS-DA analysis (Figure S2A). Six of these lipoprotein variables, including VLDL-4 *tg*, LDL-1 and LDL-2 *chol*, HDL *phol* and HDL-2 *chol*, remained significant in LASSO regression analysis. In summary, a total of 10 lipoprotein variables that were selected by two of three analyses, including logistic regression analysis, PLS-DA analysis and LASSO regression analysis, were identified as important lipoprotein variables for distinguishing NCA and UA (Figure 3A). These variables were VLDL-4 *tg*, LDL-1 *chol*, five LDL-2 particles, HDL *phol*, HDL-2 *chol* and HDL-3 Apo-A1 (Table S3).

**Figure 4:**
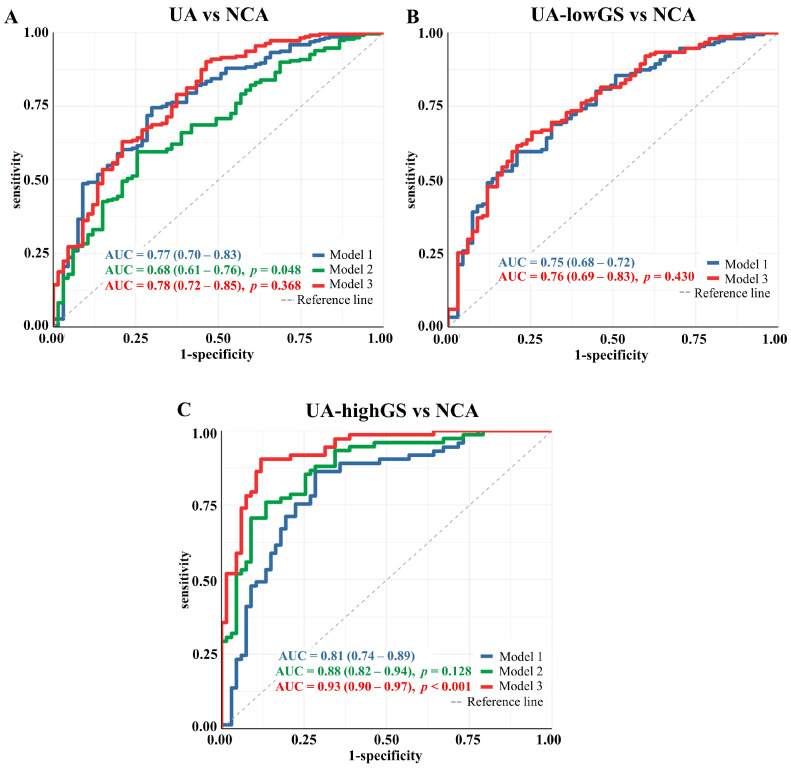
Performance of the selected lipoprotein variables in unstable angina (UA) discrimination based on Logistic Regression models. Area under the curve (AUC) calculated from receiver operating characteristic (ROC) of pairwise discrimination of UA patients and patients in two UA subgroups from those with normal coronary arteries (NCA) in logistic regression models, respectively. These logistic regression models are applied for measuring the added value of the selected variables for discriminating (**A**) UA patients, (**B**) UA patients with a low Gensini score (UA-lowGS, GS ≤ 25.4) and (**C**) UA patients with a high Gensini score (UA-highGS, GS ≤ 25.4) from the NCA group. In model 1, the predictive value of sex, age, body mass index and use of lipid-lowering drugs was included. In model 2, the selected discriminating lipoprotein variables for two patient groups were included. Ten lipoprotein variables were selected in the comparison of UA and NCA and thirty-two variables in the comparison of highGS and NCA. In the comparison of lowGS and NCA, there was only one lipoprotein variable was selected, so no Model 2 was built for discriminating these two groups. In model 3, four risk factors from model 1 and selected lipoprotein variables from model 2 were added.

Further, we tested if the selected lipoprotein variables can predict the risk of UA through logistic-regression-based discrimination (UA versus NCA). Model 1 was adjusted by sex, age, body mass index (BMI) and lipid-lowering drugs and depicted an AUCROC of 0.77 (95% CI: 0.70–0.83), while model 2 included the 10 selected lipoprotein variables depicted an AUCROC of 0.68 (95% CI: 0.61–0.76). However, by adjusting the model with the ten lipoprotein variables together with sex, age and BMI, model 3 slightly improved the discrimination with an AUCROC value of 0.78 (95% CI: 0.72–0.85, *p* = 0.368) (Figure 4A).

### 3.3. Lipoprotein Particles Show Higher Performance in Discriminating UA Patients with High Gensini Scores from Angiographically Normal Coronary Arteries Patients

Seventy lipoprotein variables had significant associations with the risk of UA with highGS in logistic regression analysis, while there was no lipoprotein found significantly associated with lowGS (Figure 2). The PLS-DA analysis identified a panel of 42 lipoprotein variables in the model of lowGS and NCA groups, and 41 variables in the model of highGS and NCA groups (Figure S2B,C). Lasso regression analysis identified LDL-1 *chol* as a potential variable that can distinguish lowGS patients from NCA patients. Three LP variables were identified by Lasso regression analysis to distinguish highGS patients from NCA patients, including HDL-C, HDL *phol* and VLDL-4 *tg*. A total of 32 lipoprotein variables, 29 of them selected by PLS-DA and LASSO, and 3 LPs selected by logistic regression, PLS-DA and LASSO, were identified as distinguishing lipoprotein variables from highGS patients to NCA patients (Figure 3C). The selected LP variables are listed in Table S3. Only LDL-1 *chol* was selected by all three methods and was identified as a distinguishing variable for lowGS patients (Figure 3B). No improvement was observed in the discrimination of lowGS and NCA patients after adding LDL-1 *chol* to the four non-lipid risk factors of model 1 (Figure 4B). However, adding these 32 selected lipoprotein variables into model 3 for discriminating highGS and NCA patients, we observed significant improvement in the discrimination with an increasing AUC by 14.9% when compared to model 1 (AUC = 0.93 [95% CI: 0.90–0.97] vs. AUC = 0.81 [95% CI: 0.74–0.89], *p* < 0.01) (Figure 4C).

### 3.4. Sensitivity Analysis

During sensitivity analysis, a total of 223 patients (NCA = 61, lowGS = 106, highGS = 56) who did not receive lipid-lowering drugs at baseline were included. The analysis was applied to control the effect of using lipid-lowering drugs. The associations of lipoprotein variables with UA, lowGS and highGS compared to NCA patients were tested by logistic regression analysis. The number of significant LP variables in the comparison of highGS and NCA patients was reduced but there were still 33 variables that remained significant. Most of them were VLDL variables and triglycerides from different LP particles (Figure S3). However, no LP variable was found that had a significant association with the risk of UA or lowGS compared to NCA.

### 3.5. The Associations between Lipoproteins and Clinical Biomarkers of Cardiovascular Diseases

To investigate the distribution of LPs in blood and the potential biological pathways involved in coronary atherosclerosis, 112 LP variables were further analyzed to investigate their correlations with six CVD biomarkers (glucose, HbA1c, creatinine, hs-CRP, fibrinogen and hs-TnT) and the Gensini score.

**Figure 5:**
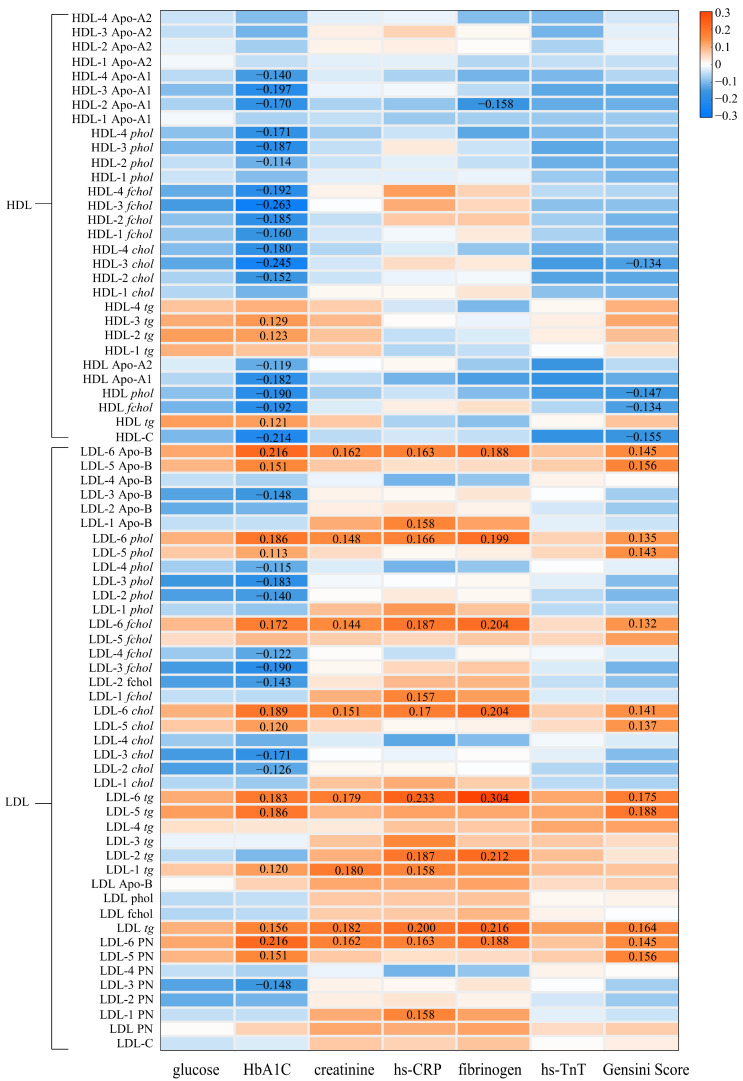

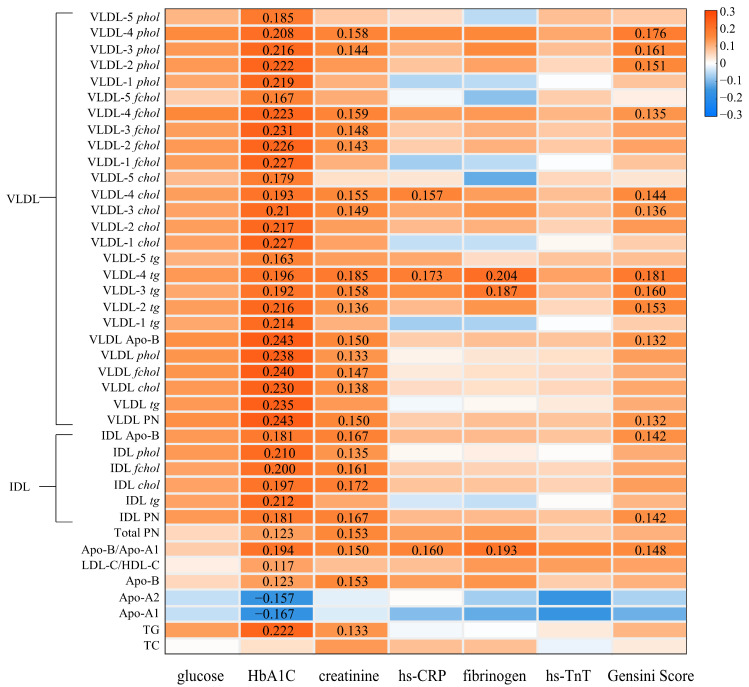
Partial Correlation Coefficients (R) between Lipoprotein Particles and Atherosclerosis-related Biomarkers and Gensini Score. Partial correlations were adjusted for sex, age, body mass index and the use of lipid-lowering drugs. The correlation coefficient values that were significant (FDR-p value < 0.1) are shown in the heatmap. Apo-A1, apolipoprotein A1; Apo-A2, apolipoprotein A2; Apo-B, apolipoprotein B; *chol*, cholesterol; *fchol*, free cholesterol; HbA1c, glycosylated haemoglobin; HDL-C, high-density lipoprotein cholesterol; hs-CRP, high-sensitivity C-reactive protein; hs-TnT, high-sensitivity Troponin T; IDL-C, intermediate-density lipoprotein cholesterol; LDL-C, low-density lipoprotein cholesterol; *phol*, phospholipids; PN, particle number; TC, total cholesterol; TG, total triglycerides; *tg*, triglycerides; VLDL-C, very low-density lipoprotein cholesterol.

Figure 5 shows that no LP variables were found to be significantly correlated with glucose and hs-TnT after controlling confounders and multiple comparison corrections (FDR-*p* < 0.1). After adjusting for sex, age, BMI and the use of lipid-lowering drugs, the number of LP variables that were significantly associated with HbA1c, creatinine, hs-CRP and fibrinogen and the Gensini scores were 83, 28, 4, 12 and 21, respectively (Figure 5). As for HbA1c, the results showed that 53 lipoprotein variables, including IDL, VLDL main fraction and subfractions, LDL-5 and 6 subfractions, and *tg* in HDL, HDL-2 and 3, had positive correlations with HbA1c (r: 0.113 to 0.243, FDR-p < 0.1); conversely, 30 variables, which mainly were Apo-A1, Apo-A2, LDL-2 and 3, HDL main fraction and subfractions, had negative correlations with it (r: −0.114 to −0.263, FDR-p < 0.1). Creatinine had weak but significant positive correlations with Apo-B, the ratio of Apo-B/Apo-A1 and total particle number (r: 0.150 to 0.153, FDR-p < 0.1). Moreover, creatinine level was positively associated with the LPs containing Apo-B, including IDL, VLDL (VLDL-3 and VLDL-4) and LDL (LDL-6) particles (r: 0.138 to 0.185, FDR-p < 0.1). Fibrinogen and hs-CRP showed positive correlations with fewer lipoprotein variables, most of which were LDL-6 variables. The Gensini score had positive associations with IDL, VLDL subfractions (VLDL-2, 3 and 4) and small LDL subfractions (LDL-5 and 6) (r: 0.141 to 0.188, FDR-p < 0.1), and negative associations with HDL-C and HDL *phol* (r: −0.155 to −0.147, FDR-p < 0.1).

Notably, Apo-B/Apo-A1 ratio, VLDL-3 *tg*, VLDL-4 *tg* and LDL-6 *tg* showed positive correlations with more CVD biomarkers and the Gensini score than other variables, indicating that these lipoprotein variables may be important factors reflecting the degree of coronary stenosis in UA patients.

## 4. Discussion

The study proves that coronary lesions are associated with a change in concentrations of human blood plasma lipoproteins in unstable angina patients. We demonstrated that a plasma lipoprotein particle profile measured by ^1^H NMR could be used to distinguish UA patients with high Gensini score (>25.4) from the NCA group with a satisfactory discrimination accuracy. This may offer a strategy to classify patients with different severities of coronary lesions according to their lipoprotein profile.

Previous studies showed that LDL subfractions with different sizes play dissimilar roles in CVD, and their associations with CVD are inconsistent. Small dense LDL particles (sdLDL) are more atherogenic and associated with higher mortality in patients with MI [36]. Large LDL particles, which were generally considered less non-atherogenic, have positive associations with the risk of death in patients who underwent coronary angiography [19]. However, another study found that LDL-2 particles, which were identified as a large LDL subfraction, had a positive association with the large HDL subfraction that had a protective effect on CVD in the atherogenic group [18]. This is in accordance with our results that LDL-2 variables were lower in UA patients and showed positive associations with UA. However, the association between UA and small LDL subclasses (LDL-6) with a density around 1.044–1.063 kg/L [37] was not observed in our study. This is in agreement with previous studies suggesting that there are discontinuities across the LDL subclasses indicative of subclass-specific lipoprotein metabolism [38].

Our results also prove that more serious coronary lesions (high-GS) is associated with plasma concentrations of VLDL, LDL, and HDL main and subfractions, including molecular classes such as triglycerides and Apo-B/Apo-A1 ratio. Among these LP variables, VLDL particles are lipoproteins containing Apo-B, and HDL particles are rich in Apo-A1, so Apo-B/Apo-A1 is a response variable reflecting the changes in VLDL and HDL particles and can be a potential biomarker for highGS. The results of the sensitivity analysis showed after removing the patients with lipid-lowering treatment, 42.8% of LP variables (33 out of 70 variables) remained significant in the comparison of highGS and NCA groups, suggesting that the LP profiles between these two groups are a result of the severity of coronary lesions.

The LP profiles between lowGS and highGS patients were different, indicating that these two groups of UA patients have different underlying pathophysiology and respond differently to medical therapies. In this study, the severity of coronary lesions of lowGS and highGS differed greatly. The average Gensini score of the former group was four times lower than that of the latter, and no patient in the lowGS group had obstruction lesions. Some studies have shown that coronary heart disease patients with a Gensini score below 25 have a low occurrence of cardiovascular events [39]. Therefore, this might be the reason why the LP profiles in the lowGS group and patients without coronary stenosis (NCA) in this study were more similar.

In the correlation analysis between LP and CVD-related biomarkers, no LP variables were found to be significantly correlated with glucose, hs-TnT and Gensini score. However, before multiple comparison corrections, Apo-B/Apo-A1, LDL-5 and 6, VLDL-3 and 4 were positively correlated with the Gensini score. Results from previous studies investigating the correlations between LPs and Gensini score have been conflicting. One study reveals that large size and small size HDL-C had opposite correlation with the Gensini score in patients with angina [40], while some other studies show that the HDL subfraction and sdLDL are correlated with the Gensini score [41]. More population studies are needed to validate the association and mechanism between LP and Gensini score.

Regarding HbA1c, the positive correlations between 26 VLDL variables and HbA1c may be related to insulin resistance, which is usually accompanied by high levels of blood glucose and HbA1c in diabetes patients. In addition, long-term hyperglycemia contributes to the glycosylation of LDL particles. This glycosylation process mainly influences Apo-B and phospholipids of LDL, which decreases the LDL clearance and increases the LDL oxidation susceptibility [42]. The present study found that 28 LP variables were positively correlated to creatinine, most of which were Apo-B-rich LPs such as IDL, VLDL and small LDL particles. An increased level of creatinine indicates a disorder of kidney function. Patients with nephrotic syndrome usually have higher plasma levels of cholesterol, triglycerides, Apo-B-containing LP and lipoprotein(a) [43]. The underlying molecular mechanism between creatinine and LPs remained unclear. Instead of creatinine, some studies observed the correlation between urinary albumin-to-creatinine ratio (UACR) and LPs. Recently, a cross-sectional study based on 35,751 Chinese participants found that TG/HDL-C had significant associations with UACR, while other LPs or the ratio between TG and other LPs were not [44]. However, a Mendelian randomization study suggested that higher TG and LDL-C caused the elevation of UACR [45]. Due to the inconsistency of related research, the underlying molecular mechanism between creatinine and LPs still needs further investigation. Only 4 LDL triglyceride variables were positively correlated with hs-CRP. This relationship could be explained by the fact that CRP directly binds to highly oxidized LDL-C in lipid-laden plaques [46]. Fibrinogen, as a coagulation/inflammatory biomarker strongly associated with atherogenesis [47], was correlated with Apo-B/Apo-A1, LDL-6 particles, VLDL-3 *tg* and VLDL-4 *tg*, suggesting that LPs play a prominent role in the thrombosis or inflammation process of unstable angina.

Twenty-one LPs showed significant correlations with the Gensini score, mainly including VLDL and LDL-5 and 6 particles. VLDL particles play an important role in the development of atherosclerosis via the mechanism of endothelial dysfunction and the increase of systemic inflammation [48]. In addition, macrophages take up VLDL particles directly and change them into foam cells, which increases cholesterol accumulation in the intima and atherosclerotic plaque formation [21]. Regarding LDL, prior studies showed that sdLDL [49], which has a similar size to LDL-6 [50], is an emerging cardiovascular risk factor and an atherogenic LP [51]. Our study showed a similar finding that LDL-6 particles had more positive correlations with the above-mentioned CVD biomarkers and Gensini score.

In clinical practice, coronary angiography is a reliable tool for detecting the severity of coronary lesions in UA patients [2]. However, this expensive invasive test is not acceptable for some patients. Additionally, it is unnecessary for every patient with angina, because 59% of patients who undergo coronary angiography are found to have no obstructed coronary arteries [52]. Therefore, the identification of patients with severe coronary lesions early using non-invasive techniques will allow researchers to develop better treatment strategies and health status improvement. Our study showed that the integration of lipoprotein particles assays with cardiovascular risk factors has the potential to improve the identification of the degree of coronary lesions in patients with unstable angina at an early stage without using unnecessary therapies and resources. Comprehensive lipoprotein profiling may provide a classification of lipoprotein pathophysiology that could help to understand the inconsistent associations between the concentration of LDL-C and the high risk of CVD or all-cause mortality [8], and lead to more effective management of CVD risk in patients.

Although we identified differing lipoprotein profiles between lowGS and highGS patients, it does not prove a causal role of a change of lipoprotein profiles in as a result of the progression of coronary lesions. External validation and longer follow-ups are needed to confirm and extend our findings by providing more conclusive evidence regarding the causal associations between LPs and UA. To reflect the disease progression and prognosis in patients with unstable angina more comprehensively, further study is needed to investigate the correlation of lipoprotein profile and other characteristics of atherosclerotic plaque in unstable angina patients, such as the vulnerability and activity evaluated by Optical Coherence Tomography (OCT) or Intravascular ultrasound (IVUS).

## 5. Conclusions

This is the first study investigating the relationships between lipoprotein main/subfractions and coronary stenosis in unstable angina patients. Increased VLDL-4 *tg* and decreased LDL-1 *chol*, HDL *phol*, HDL-2 *chol* and HDL-3 Apo-A1 concentrations as well as LDL-2 particle numbers (incl. *fchol* and *chol*, *phol*, Apo-B) were found to be key LP variables distinguishing UA from NCA patients. Increased Apo-B/Apo-A1, VLDL particles and decreased Apo-A1, plasma LDL particles (LDL-2 and LDL-3 *chol*, *fchol* and *phol*), HDL particles (HDL main fraction, HDL-1 and HDL-2 *chol*, *fchol*, *phol*, and Apo-A1) are characteristics of UA patients with a high Gensini score that distinguishes them from NCA patients. Thus, the findings from our study prove that blood plasma lipoproteins can be used as biomarkers to distinguish UA patients with severe coronary lesions from NCA patients.

## Figures and Tables

**Table 1:** Characteristics of the study patients.

	NCA (n = 67)	UA (n = 230)	*p* ^1^	LowGS (n = 155)	HighGS (n = 75)	*p* ^2^
Age, years	61.6 ± 7.25	65.35 ± 7.25	<0.01	65.14 ± 7.76	65.77 ± 6.11	0.81
Female	43 (64.2)	85 (37.0)	<0.01	66 (42.6)	19 (25.3)	0.01
BMI, kg/m^2^	23.3 ± 3.92	24.34 ± 2.87	0.04	24.21 ± 2.82	24.62 ± 2.97	0.63
Blood Pressure, mmHg						
Systolic	128.91 ± 21.29	132.97 ± 20.25	0.11	131.5 ± 19.78	136 ± 21.01	0.26
Diastolic	76.82 ± 9.92	79.43 ± 11.38	0.20	79.11 ± 11.41	80.08 ± 11.36	0.81
Current smoker	9 (13.4)	64 (27.8)	0.04	42 (27.1)	22 (29.3)	0.57
Comorbidity						
Hypertension	28 (41.8)	139 (60.4)	0.02	93 (60.0)	46 (61.3)	0.85
Type 2 diabetes	7 (10.5)	56 (24.4)	<0.01	32 (20.7)	24 (32.0)	0.06
Lipid-lowering drugs	6 (9.0)	68 (29.6)	<0.01	49 (31.6)	19 (25.3)	0.33
Gensini score	0	25.4 ± 25.16	<0.01	12.36 ± 6.61	52.35 ± 27.87	<0.01
Number of affected arteries			<0.01			<0.01
0	67 (100)	7 (3.0)		7 (4.5)	0 (0)	
1	0	42 (18.3)		39 (25.2)	3 (4.0)	
2	0	72 (31.3)		54 (34.8)	18 (24.0)	
3	0	109 (47.4)		55 (35.5)	54 (72.0)	
Stenosis location						
Left main artery	0	23 (10.0)	<0.01	4 (2.6)	19 (25.3)	<0.01
Left anterior descending artery	0	205 (89.1)	<0.01	133 (85.8)	72 (96.0)	0.02
Circumflex coronary artery	0	140 (60.9)	<0.01	74 (47.7)	66 (88.0)	<0.01
Right coronary artery	0	168 (73.0)	<0.01	105 (67.7)	63 (84.0)	<0.01
Laboratory data						
ALT, units/L	20.15 ± 8.77	22.71 ± 12.49	0.25	23.06 ± 13.58	21.95 ± 9.83	0.80
AST, units/L	19.94 ± 7.01	22.03 ± 11.59	0.27	22.45 ± 11.65	21.15 ± 11.5	0.69
Total cholesterol, mmol/L	4.75 ± 1.03	4.38 ± 1.08	0.05	4.36 ± 1.07	4.43 ± 1.10	0.88
Triglycerides, mmol/L	1.40 ± 0.71	1.65 ± 1.07	0.19	1.63 ± 1.20	1.68 ± 0.74	0.94
HDL-C, mmol/L	1.21 ± 0.30	1.06 ± 0.26	<0.01	1.11 ± 0.27	0.96 ± 0.19	<0.01
LDL-C, mmol/L	2.92 ± 0.76	2.71 ± 0.83	0.10	2.67 ± 0.82	2.81 ± 0.85	0.48
Apo-A1, g/L	1.26 ± 0.22	1.20 ± 0.21	<0.01	1.23 ± 0.21	1.14 ± 0.20	<0.01
Apo-B, g/L	0.84 ± 0.20	0.83 ± 0.23	0.10	0.80 ± 0.23	0.87 ± 0.22	0.08
Apo-E, mg/L	42.2 ± 9.97	39.67 ± 11.04	0.15	40.18 ± 11.48	38.55 ± 10.01	0.56
Blood glucose, mmol/L	4.97 ± 0.96	5.61 ± 1.81	<0.01	5.45 ± 1.64	5.96 ± 2.10	0.08
HbA1c, %	5.75 ± 0.84	6.21 ± 1.26	<0.01	6.03 ± 0.93	6.59 ± 1.71	<0.01
creatinine, μmol/L	77.51 ± 14.88	85.63 ± 16.4	<0.01	83.53 ± 15.92	90.09 ± 16.61	0.01
hs-CRP, mg/L	2.85 ± 5.47	4.6 ± 9.70	0.35	4.43 ± 10.10	4.95 ± 8.85	0.92
Fibrinogen, g/L	3.07 ± 0.82	3.17 ± 0.89	0.05	3.07 ± 0.78	3.36 ± 1.06	0.06
hs-TnT, ng/L	9.47 ± 9.88	15.53 ± 27.3	<0.01	9.86 ± 8.09	27.25 ± 44.35	<0.01

Values are mean ± SD or n (%). ALT, alanine transaminase; Apo-A1, apolipoprotein A1; Apo-B, apolipoprotein B; Apo-E, apolipoprotein E; AST, aspartate aminotransferase; BMI, body mass index; GS, Gensini score; HbA1c, glycosylated hemoglobin; HDL-C, high-density lipoprotein cholesterol; hs-CRP, high-sensitivity C-reactive protein; hs-TnT, high-sensitivity Troponin T; LDL-C, low-density lipoprotein cholesterol; NCA, normal coronary artery; UA, unstable angina. ^1^ *p* value for the comparison between NCA and UA patients. ^2^ *p* value for the comparison within UA subgroups.

## Data Availability

Data will be made available upon reasonable request.

## Supplementary Materials

The following supporting information can be downloaded at: https://www.mdpi.com/article/10.3390/metabo13020273/s1, Figure S1: ASCA analysis of the effect of specific covariables on lipoprotein variation of patients with unstable angina (UA) and those with normal coronary arteries (NCA); Figure S2: The variable importance in projection (VIP) score plot of 112 lipoprotein variables in partial least squares-discriminant analysis (PLS-DA) of three pairwise comparisons: (A) unstable angina (UA) and patients with normal coronary arteries (NCA), (B) UA patients with low Gensini score (UA-lowGS, GS ≤ 25.4) and NCA group, (C) UA patients with high Gensini score (UA-highGS, GS ≤ 25.4) and NCA group; Figure S3: Associations of 112 Lipoprotein Variables with Unstable Angina (UA) in the UA patients without lipid-lowering treatment at baseline (n = 223); Table S1: Correlation coefficients of five lipoproteins measured by clinical chemistry and NMR spectroscopy; Table S2: Lipoprotein variables with median concentrations (interquartile range (IQR, 25th–75th percentile)) in different groups, and reported FDR-corrected *p*-value, fold change for pairwise comparisons; Table S3: List of discriminating variables selected as discriminating lipoprotein variables to classify UA, lowGS, highGS and NCA in a pairwise model.
