# Supplementary material for: Alterations of NMR-Based Lipoprotein Profile Distinguish Unstable Angina Patients with Different Severity of Coronary Lesions
Source: Metabolites. 2023 Feb 14;13(2):273. doi: 10.3390/metabo13020273 (PMC9958945; doi:10.3390/metabo13020273)
Supplement: Supplementary file 1 [file metabolites-13-00273-s001.zip › metabolites-1910481-supplementary.pdf]

Alterations of NMR-Based Lipoprotein Profile Distinguish Unstable Angina Patients with Different Severity of Coronary Lesions

Yongxin Ye [1,2,†], Jiahua Fan [1,2,†], Zhiteng Chen [3], Xiuwen Li [4], Maoxiong Wu [3], Wenhao Liu [3], Shiyi Zhou [1,2], Morten Arendt Rasmussen [5,6], Søren Balling Engelsen [5], Yangxin Chen [3], Bekzod Khakimov [5,*] and Min Xia [1,2,*]

[1] Department of Nutrition, School of Public Health, Sun Yat-sen University (Northern Campus), Guangzhou 510080, China
[2] Guangdong Provincial Key Laboratory of Food, Nutrition and Health, Guangzhou 510080, China
[3] Department of Cardiology, Sun Yat-Sen Memorial Hospital, Sun Yat-sen University, Guangzhou 510120, China
[4] Department of Medical Statistics and Epidemiology, School of Public Health, Sun Yat-sen University, Guangzhou 510080, China
[5] Department of Food Science, University of Copenhagen, 1958 Frederiksberg C, Denmark
[6] COPSAC—Copenhagen Prospective Studies on Asthma in Childhood, Herlev and Gentofte Hospital, University of Copenhagen, 2100 Copenhagen, Denmark
* Correspondence: bzo@food.ku.dk (B.K.); xiamin@mail.sysu.edu.cn (M.X.); Tel.: +45-35328184 (B.K.); +86-(020)-87332433 (M.X.)
† These authors contributed equally to this work.

Table S1. Correlation coefficients of five lipoproteins measured by clinical chemistry and NMR spectroscopy.

Trait	n*	Mean (standard deviation)		Correlation coefficient	P value
		Clinical chemistry	NMR spectroscopy		
Total cholesterol (mmol/L)	283	4.47 (1.07)	4.35 (1.00)	0.923	<0.01
Triglycerides (mmol/L)	283	1.59 (1.00)	1.47 (0.80)	0.926	<0.01
LDL-cholesterol (mmol/L)	283	2.76 (0.82)	2.15 (0.76)	0.806	<0.01
HDL-cholesterol (mmol/L)	283	1.10 (0.28)	1.23 (0.26)	0.934	<0.01
ApoA1 (g/L)	283	1.22 (0.22)	1.27 (0.17)	0.851	<0.01
ApoB (g/L)	283	0.83 (0.22)	0.75 (0.21)	0.902	<0.01

* 283 patients who had the value of lipoproteins measured by clinical chemistry were included in the correlation analysis of lipoproteins measured by clinical chemistry and NMR spectroscopy. ** To convert cholesterol (mg/dL) values to standard international units (mmol/L), multiply by 0.0259; to convert triglycerides to mmol/L, multiply by 0.0113; for ApoB and ApoA1, convert to g/L by multiplying by 0.01. Abbreviation: ApoB, Apolipoprotein B; ApoA1, Apolipoprotein A; NMR, nuclear magnetic resonance.

Table S2. Lipoprotein variables with median concentrations (interquartile range (IQR, 25th-75th percentile)) in different groups, and reported FDR-corrected p-value, fold change for pairwise comparisons.

Lipoprotein variables	Unit	Median (IQR)				UA/NCA	low GS/NCA	high GS/NCA
		NCA (n=67)	UA (n=230)	Low GS (n=155)	High GS (n=75)	Fold change	Fold change	Fold change
TC	mg/dL	175.86 (156.45, 196.84)	161.35 (136.54, 189.97)	160.94 (136.59, 190.24)	161.63 (136.69, 189.88)	0.92	0.92	0.92
TG	mg/dL	99.33 (81.42, 139.16)	113.27 (85.14, 160.61)	108.33 (77.8, 159.89)	121.31 (94.65, 160.8)	1.14	1.09	1.22
LDL-C	mg/dL	85.76 (66.33, 104.66)	77.59 (61.43, 98.6)	76.25 (60.32, 98.94)	79.95 (66.27, 95.76)	0.90	0.89	0.93
HDL-C	mg/dL	49.37 (42.66, 57.24)	45.45 (39.12, 51.35)	47 (40.36, 53.27)	41.82 (37.8, 47.36)	0.92	0.95	0.85
Apo-A1	mg/dL	130.94 (120.8, 143.18)	123.67 (113.48, 135.54)	128.06 (116.29, 137.93)	119.63 (108.66, 129.86)	0.94	0.98	0.91
Apo-A2	mg/dL	24.91 (21.97, 28.33)	24.29 (21.67, 26.77)	24.76 (22.26, 27.17)	23.37 (21.11, 25.81)	0.97	0.99	0.94
Apo-B	mg/dL	74.53 (64.33, 87.94)	71.75 (59.18, 88.95)	71.36 (57.07, 86.31)	74.08 (65.66, 93.03)	0.96	0.96	0.99
LDL-C/HDL-C	-/-	1.74 (1.41, 2.12)	1.73 (1.33, 2.19)	1.64 (1.25, 2.14)	1.88 (1.49, 2.24)	0.99	0.94	1.08
Apo-B/Apo-A1	-/-	0.56 (0.49, 0.7)	0.58 (0.48, 0.71)	0.56 (0.45, 0.7)	0.66 (0.54, 0.75)	1.04	1.00	1.18
Total Particle Number	nmol/L	1355.18 (1169.61, 1598.99)	1304.46 (1076.14, 1617.43)	1297.55 (1037.7, 1569.26)	1347 (1193.91, 1691.61)	0.96	0.96	0.99
VLDL Particle Number	nmol/L	142.52 (107.66, 189.81)	163.05 (120.36, 218.03)	143.92 (110.91, 215.53)	172.44 (136.09, 237.68)	1.14	1.01	1.21
IDL Particle Number	nmol/L	58.59 (41.44, 83.69)	62.69 (42.25, 89.65)	59.5 (39.1, 86.78)	67.53 (53.64, 97.26)	1.07	1.02	1.15
LDL Particle Number	nmol/L	1059.04 (906.08, 1320.77)	1003.57 (817.44, 1295.55)	960.83 (782.63, 1233.63)	1025.83 (906.28, 1335.68)	0.95	0.91	0.97
LDL-1 Particle Number	nmol/L	203.99 (169.84, 246.41)	179.08 (147.48, 221.41)	173.92 (145.77, 215.23)	182.53 (155.79, 227.18)	0.88	0.85	0.89
LDL-2 Particle Number	nmol/L	148.55 (116.31, 177.75)	122.3 (95.95, 153.06)	122.89 (96.33, 151.82)	121.16 (91.03, 155.17)	0.82	0.83	0.82
LDL-3 Particle Number	nmol/L	118.53 (83.46, 154.11)	100.55 (60.08, 141.16)	104.39 (63.07, 142.1)	96.15 (57.77, 128.66)	0.85	0.88	0.81
LDL-4 Particle Number	nmol/L	109.76 (49.81, 148.42)	88.61 (46.3, 142.88)	84.23 (35.69, 155.65)	89.02 (62.45, 134.55)	0.81	0.77	0.81
LDL-5 Particle Number	nmol/L	141.74 (99.02, 228.8)	156.07 (103.7, 223.56)	149.01 (96.83, 209.57)	175.83 (122.14, 242.51)	1.10	1.05	1.24
LDL-6 Particle Number	nmol/L	302.12 (225.66, 412.87)	322.77 (243.86, 440.62)	309.15 (235.4, 429.92)	341.88 (263.13, 458.91)	1.07	1.02	1.13
IDL Triglycerides	mg/dL	7.37 (3.78, 13.62)	9.04 (4.59, 16.4)	7.83 (3.42, 15.97)	10.67 (5.71, 17.26)	1.23	1.06	1.45
LDL Triglycerides	mg/dL	16.49 (13.16, 19.22)	16.05 (13.35, 19.98)	15.25 (12.39, 19.18)	17.15 (14.54, 20.56)	0.97	0.92	1.04
HDL Triglycerides	mg/dL	11 (9.34, 13.01)	10.59 (9.01, 13.18)	10.5 (8.78, 13.18)	11.03 (9.32, 13.17)	0.96	0.95	1.00
VLDL Triglycerides	mg/dL	60.68 (41.71, 91)	72.32 (51.31, 106.8)	64.58 (43.82, 104.94)	81.17 (58.4, 107.35)	1.19	1.06	1.34

VLDL Cholesterol	mg/dL	17.64 (12.46, 29.11)	20.29 (13.79, 30.8)	19.02 (12.52, 30.76)	21.24 (16.81, 33.69)	1.15	1.08	1.20
IDL Cholesterol	mg/dL	7.85 (5.14, 12.16)	8.55 (4.92, 13.85)	7.92 (4.56, 13.19)	9.08 (6.68, 14.74)	1.09	1.01	1.16
VLDL Free Cholesterol	mg/dL	8.56 (6.42, 12.72)	9.72 (7, 13.81)	9.11 (6.51, 13.41)	10.11 (8.37, 14.57)	1.14	1.06	1.18
IDL Free Cholesterol	mg/dL	2.31 (1.57, 3.59)	2.62 (1.48, 4.14)	2.47 (1.26, 3.99)	2.8 (1.96, 4.33)	1.13	1.07	1.21
LDL Free Cholesterol	mg/dL	25.29 (19.96, 28.67)	21.99 (17.37, 27.86)	21.98 (16.85, 27.97)	22.09 (18.5, 27.7)	0.87	0.87	0.87
HDL Free Cholesterol	mg/dL	11.02 (8.95, 13.11)	9.42 (7.52, 11.38)	10.05 (7.88, 11.81)	8.54 (7.36, 10.2)	0.85	0.91	0.77
VLDL Phospholipids	mg/dL	17.62 (13.19, 25.48)	20.66 (15.47, 29.19)	18.87 (14.07, 27.89)	22.5 (18.06, 30.1)	1.17	1.07	1.28
IDL Phospholipids	mg/dL	4.48 (3, 7.28)	4.88 (2.75, 7.88)	4.5 (2.08, 8.03)	5.08 (3.33, 7.68)	1.09	1.00	1.13
LDL Phospholipids	mg/dL	51.45 (41.28, 59.71)	45.82 (38.31, 56.57)	44.98 (37.9, 56.84)	47.26 (40.82, 54.86)	0.89	0.87	0.92
HDL Phospholipids	mg/dL	70.86 (60.96, 79.08)	63.21 (56.47, 70.43)	65.24 (57.49, 71.8)	59.13 (53.12, 66.95)	0.89	0.92	0.83
HDL Apo-A1	mg/dL	130.51 (117.69, 141.66)	122.28 (110.46, 134.78)	125.33 (112.58, 138.56)	117.76 (105.63, 128.41)	0.94	0.96	0.90
HDL Apo-A2	mg/dL	25.72 (22.96, 29.02)	25.44 (22.75, 27.66)	25.74 (23.32, 28.15)	24.05 (22.28, 26.94)	0.99	1.00	0.94
VLDL Apo-B	mg/dL	7.84 (5.92, 10.44)	8.97 (6.62, 11.99)	7.92 (6.1, 11.85)	9.48 (7.48, 13.07)	1.14	1.01	1.21
IDL Apo-B	mg/dL	3.22 (2.28, 4.61)	3.45 (2.32, 4.93)	3.27 (2.16, 4.77)	3.71 (2.95, 5.35)	1.07	1.02	1.15
LDL Apo-B	mg/dL	58.24 (49.84, 72.64)	55.2 (44.96, 71.25)	52.84 (43.04, 67.85)	56.42 (49.85, 73.46)	0.95	0.91	0.97
VLDL-1 Triglycerides	mg/dL	24.09 (15.53, 44.13)	32.57 (20.44, 57.98)	29.41 (18.03, 58.28)	39.46 (22.95, 56.33)	1.35	1.22	1.64
VLDL-2 Triglycerides	mg/dL	9.15 (5.55, 16.2)	10.86 (6.59, 17.53)	9.52 (6.01, 17.26)	12.25 (9.02, 18.4)	1.19	1.04	1.34
VLDL-3 Triglycerides	mg/dL	8.79 (4.8, 12.63)	10.14 (6.39, 14.76)	8.82 (5.3, 13.99)	11.22 (8.07, 16.14)	1.15	1.00	1.28
VLDL-4 Triglycerides	mg/dL	7.92 (5.64, 10.17)	8.94 (6.57, 11.66)	8.51 (6.1, 10.95)	9.86 (8.02, 13.06)	1.13	1.07	1.24
VLDL-5 Triglycerides	mg/dL	3.18 (2.72, 3.64)	3.18 (2.76, 3.67)	3.07 (2.75, 3.59)	3.42 (2.91, 3.75)	1.00	0.97	1.08
VLDL-1 Cholesterol	mg/dL	5.95 (3.56, 9.79)	6.96 (4.39, 11.5)	6.31 (3.96, 11.49)	7.49 (5.03, 11.44)	1.17	1.06	1.26
VLDL-2 Cholesterol	mg/dL	2.29 (1.4, 4.18)	2.7 (1.64, 4.48)	2.43 (1.37, 4.43)	3.14 (2.15, 4.56)	1.18	1.06	1.37
VLDL-3 Cholesterol	mg/dL	2.61 (1.5, 4.63)	3.18 (1.78, 5.17)	2.8 (1.33, 4.97)	3.69 (2.57, 5.72)	1.22	1.07	1.41
VLDL-4 Cholesterol	mg/dL	4.4 (2.7, 6.1)	4.65 (2.99, 6.64)	4.26 (2.69, 6.53)	5.36 (3.98, 7.64)	1.06	0.97	1.22
VLDL-5 Cholesterol	mg/dL	2.04 (1.66, 2.42)	2.08 (1.73, 2.43)	2.01 (1.69, 2.41)	2.16 (1.86, 2.55)	1.02	0.99	1.06
VLDL-1 Free Cholesterol	mg/dL	2.02 (1.01, 3.63)	2.52 (1.47, 4.37)	2.25 (1.15, 4.58)	2.74 (1.69, 4.3)	1.25	1.11	1.36
VLDL-2 Free Cholesterol	mg/dL	1.17 (0.67, 1.83)	1.35 (0.84, 2.18)	1.21 (0.71, 2.16)	1.46 (1.07, 2.24)	1.15	1.03	1.25
VLDL-3 Free Cholesterol	mg/dL	1.19 (0.61, 1.99)	1.53 (0.9, 2.56)	1.4 (0.73, 2.48)	1.71 (1.23, 2.68)	1.28	1.18	1.44
VLDL-4 Free Cholesterol	mg/dL	1.62 (1.05, 2.44)	1.85 (1.2, 2.88)	1.68 (1.05, 2.78)	2.13 (1.53, 3.18)	1.14	1.04	1.31
VLDL-5 Free Cholesterol	mg/dL	0.84 (0.64, 1.01)	0.81 (0.61, 1.05)	0.75 (0.57, 1.01)	0.89 (0.76, 1.07)	0.96	0.89	1.06

VLDL-1 Phospholipids	mg/dL	4.54 (2.25, 7.52)	5.49 (3.34, 9.83)	5.19 (2.78, 9.84)	6.58 (3.83, 9.33)	1.21	1.14	1.45
VLDL-2 Phospholipids	mg/dL	2.54 (1.65, 4.33)	2.98 (2.05, 4.72)	2.73 (1.64, 4.69)	3.48 (2.57, 4.91)	1.17	1.07	1.37
VLDL-3 Phospholipids	mg/dL	2.97 (1.86, 4.67)	3.59 (2.36, 5.39)	3.27 (1.97, 5.33)	3.95 (3.14, 5.62)	1.21	1.10	1.33
VLDL-4 Phospholipids	mg/dL	4 (3.14, 5.17)	4.52 (3.39, 5.96)	4.16 (3.19, 5.76)	4.99 (4.12, 6.61)	1.13	1.04	1.25
VLDL-5 Phospholipids	mg/dL	2.31 (2.04, 2.74)	2.36 (2.05, 2.82)	2.29 (1.96, 2.77)	2.57 (2.18, 2.98)	1.02	0.99	1.11
LDL-1 Triglycerides	mg/dL	5.36 (4.46, 6.74)	5.43 (4.34, 7)	5.18 (3.94, 6.86)	5.69 (4.91, 7.17)	1.01	0.97	1.06
LDL-2 Triglycerides	mg/dL	2.06 (1.57, 2.54)	1.82 (1.48, 2.2)	1.78 (1.43, 2.11)	1.95 (1.53, 2.35)	0.88	0.86	0.95
LDL-3 Triglycerides	mg/dL	2.1 (1.73, 2.58)	1.96 (1.55, 2.4)	1.9 (1.55, 2.31)	2.06 (1.53, 2.55)	0.93	0.90	0.98
LDL-4 Triglycerides	mg/dL	1.59 (0.97, 2.06)	1.6 (1.07, 2.21)	1.54 (0.89, 2.1)	1.69 (1.19, 2.39)	1.00	0.97	1.06
LDL-5 Triglycerides	mg/dL	1.73 (1.15, 2.86)	2.03 (1.38, 2.92)	1.9 (1.26, 2.7)	2.38 (1.65, 3.12)	1.17	1.10	1.38
LDL-6 Triglycerides	mg/dL	3.4 (2.71, 4.33)	3.54 (2.9, 4.56)	3.38 (2.81, 4.22)	3.77 (3.26, 4.85)	1.04	0.99	1.11
LDL-1 Cholesterol	mg/dL	20.89 (17.45, 25.33)	17.63 (14.04, 22.34)	17 (13.95, 21.73)	17.97 (14.69, 22.67)	0.84	0.81	0.86
LDL-2 Cholesterol	mg/dL	14.18 (9.91, 17.03)	11 (8.29, 14.52)	11.16 (8.49, 14.53)	10.63 (7.81, 14.33)	0.78	0.79	0.75
LDL-3 Cholesterol	mg/dL	10.93 (6.95, 14.74)	8.52 (3.94, 12.73)	9.04 (4.04, 13.24)	7.73 (3.51, 10.95)	0.78	0.83	0.71
LDL-4 Cholesterol	mg/dL	9.93 (4.07, 13.18)	7.28 (3.26, 12.38)	7.38 (3.01, 12.93)	7.26 (3.79, 11.23)	0.73	0.74	0.73
LDL-5 Cholesterol	mg/dL	10.35 (7.56, 17.55)	11.71 (7.53, 16.6)	11.02 (7.07, 16.06)	12.81 (8.67, 17.97)	1.13	1.06	1.24
LDL-6 Cholesterol	mg/dL	20.14 (14.92, 25.8)	20.26 (15.78, 27.99)	19.9 (15.49, 27)	21.49 (17.33, 28.96)	1.01	0.99	1.07
LDL-1 Free Cholesterol	mg/dL	5.98 (4.81, 7.18)	5.02 (3.98, 6.47)	4.96 (3.93, 6.34)	5.38 (4.14, 6.56)	0.84	0.83	0.90
LDL-2 Free Cholesterol	mg/dL	4.06 (2.97, 5.04)	3.28 (2.24, 4.28)	3.28 (2.23, 4.33)	3.27 (2.27, 4.06)	0.81	0.81	0.81
LDL-3 Free Cholesterol	mg/dL	3.77 (2.76, 4.89)	3.11 (1.74, 4.44)	3.21 (1.85, 4.55)	2.87 (1.74, 3.95)	0.82	0.85	0.76
LDL-4 Free Cholesterol	mg/dL	3.26 (2.09, 4.17)	2.64 (1.58, 3.82)	2.71 (1.54, 3.87)	2.53 (1.91, 3.38)	0.81	0.83	0.78
LDL-5 Free Cholesterol	mg/dL	3.1 (2.09, 4.33)	3.05 (2.26, 4.35)	3 (2.09, 4.15)	3.33 (2.36, 4.67)	0.99	0.97	1.07
LDL-6 Free Cholesterol	mg/dL	4.84 (3.52, 6.1)	4.71 (3.65, 6.41)	4.63 (3.61, 6.32)	4.91 (3.73, 6.71)	0.97	0.96	1.01
LDL-1 Phospholipids	mg/dL	12 (10.07, 14.41)	10.43 (8.69, 12.83)	10.1 (8.67, 12.41)	10.61 (8.87, 13.08)	0.87	0.84	0.88
LDL-2 Phospholipids	mg/dL	7.85 (6.13, 9.77)	6.49 (5.03, 8.22)	6.61 (5.04, 8.23)	6.31 (4.75, 8.19)	0.83	0.84	0.80
LDL-3 Phospholipids	mg/dL	6.58 (4.57, 8.43)	5.27 (3.17, 7.49)	5.49 (3.32, 7.63)	4.83 (2.94, 6.66)	0.80	0.83	0.73
LDL-4 Phospholipids	mg/dL	6 (3.15, 7.65)	4.62 (2.56, 7.13)	4.65 (2.33, 7.44)	4.59 (2.95, 6.64)	0.77	0.78	0.77
LDL-5 Phospholipids	mg/dL	5.85 (4.56, 9.35)	6.54 (4.65, 8.94)	6.33 (4.35, 8.47)	7.47 (5.05, 9.96)	1.12	1.08	1.28
LDL-6 Phospholipids	mg/dL	11.13 (8.77, 14.23)	11.27 (9.18, 15.07)	11.04 (9.09, 14.79)	12 (9.6, 15.65)	1.01	0.99	1.08
LDL-1 Apo-B	mg/dL	11.22 (9.34, 13.55)	9.85 (8.11, 12.18)	9.57 (8.01, 11.84)	10.04 (8.57, 12.5)	0.88	0.85	0.89

LDL-2 Apo-B	mg/dL	8.17 (6.4, 9.78)	6.73 (5.28, 8.42)	6.76 (5.3, 8.35)	6.66 (5.01, 8.53)	0.82	0.83	0.82
LDL-3 Apo-B	mg/dL	6.52 (4.59, 8.47)	5.53 (3.3, 7.76)	5.74 (3.47, 7.82)	5.29 (3.18, 7.08)	0.85	0.88	0.81
LDL-4 Apo-B	mg/dL	6.04 (2.74, 8.16)	4.88 (2.55, 7.86)	4.63 (1.96, 8.56)	4.9 (3.43, 7.4)	0.81	0.77	0.81
LDL-5 Apo-B	mg/dL	7.8 (5.44, 12.58)	8.59 (5.7, 12.3)	8.19 (5.33, 11.53)	9.67 (6.72, 13.33)	1.10	1.05	1.24
LDL-6 Apo-B	mg/dL	16.62 (12.41, 22.71)	17.76 (13.41, 24.23)	17 (12.95, 23.65)	18.8 (14.47, 25.24)	1.07	1.02	1.13
HDL-1 Triglycerides	mg/dL	3.98 (2.89, 4.74)	3.51 (2.67, 4.48)	3.54 (2.62, 4.48)	3.49 (2.71, 4.41)	0.88	0.89	0.88
HDL-2 Triglycerides	mg/dL	1.84 (1.52, 2.34)	1.82 (1.45, 2.29)	1.77 (1.4, 2.28)	1.88 (1.49, 2.33)	0.99	0.96	1.02
HDL-3 Triglycerides	mg/dL	2.08 (1.68, 2.54)	2.05 (1.71, 2.59)	1.99 (1.64, 2.54)	2.12 (1.83, 2.72)	0.98	0.96	1.02
HDL-4 Triglycerides	mg/dL	3.39 (2.9, 3.99)	3.54 (2.91, 4.23)	3.43 (2.82, 4.22)	3.68 (3.07, 4.24)	1.04	1.01	1.09
HDL-1 Cholesterol	mg/dL	15.21 (12.51, 20.14)	13.27 (10.67, 16.31)	14.26 (11, 17.94)	12.27 (10.17, 15.02)	0.87	0.94	0.81
HDL-2 Cholesterol	mg/dL	7.53 (5.86, 9.04)	6.35 (5.33, 7.87)	6.97 (5.46, 8.17)	5.96 (4.92, 6.98)	0.84	0.93	0.79
HDL-3 Cholesterol	mg/dL	8.48 (6.81, 9.9)	7.71 (6.41, 8.6)	7.99 (6.64, 8.76)	7.1 (5.6, 8.11)	0.91	0.94	0.84
HDL-4 Cholesterol	mg/dL	16.85 (14.36, 18.95)	16.17 (13.79, 18.89)	16.5 (13.69, 19.29)	15.33 (13.84, 18.06)	0.96	0.98	0.91
HDL-1 Free Cholesterol	mg/dL	2.86 (2.12, 4.06)	2.3 (1.57, 3.19)	2.43 (1.63, 3.63)	1.93 (1.45, 2.73)	0.81	0.85	0.67
HDL-2 Free Cholesterol	mg/dL	1.22 (0.83, 1.57)	0.95 (0.6, 1.32)	1.05 (0.61, 1.47)	0.85 (0.6, 1.13)	0.77	0.86	0.70
HDL-3 Free Cholesterol	mg/dL	1.29 (0.87, 1.76)	1.11 (0.79, 1.5)	1.17 (0.84, 1.55)	1.01 (0.67, 1.22)	0.86	0.91	0.78
HDL-4 Free Cholesterol	mg/dL	2.74 (2.02, 3.21)	2.56 (1.95, 3.14)	2.61 (1.98, 3.16)	2.52 (1.81, 3.02)	0.93	0.95	0.92
HDL-1 Phospholipids	mg/dL	18.44 (14.3, 23.53)	15.68 (11.95, 19.76)	16.7 (12.38, 20.57)	14.29 (11.78, 17.11)	0.85	0.91	0.77
HDL-2 Phospholipids	mg/dL	11.8 (9.97, 14.3)	10.61 (8.35, 12.2)	11.16 (8.8, 12.8)	9.6 (8.06, 11.44)	0.90	0.95	0.81
HDL-3 Phospholipids	mg/dL	14.1 (11.67, 15.6)	12.54 (11.02, 14.27)	12.71 (11.15, 14.39)	12.01 (9.72, 13.58)	0.89	0.90	0.85
HDL-4 Phospholipids	mg/dL	23.24 (20.65, 25.71)	22.89 (20.26, 25.34)	23.08 (20.02, 25.92)	22.57 (20.4, 24.67)	0.98	0.99	0.97
HDL-1 Apo-A1	mg/dL	22.71 (16.71, 30.63)	17.81 (13.59, 24.96)	19.84 (13.6, 25.94)	16.31 (13.48, 21.24)	0.78	0.87	0.72
HDL-2 Apo-A1	mg/dL	16.01 (13.96, 17.9)	14.39 (12.12, 16.59)	14.57 (12.47, 16.96)	13.38 (10.8, 16.07)	0.90	0.91	0.84
HDL-3 Apo-A1	mg/dL	23.61 (20.26, 25.93)	21.39 (19.08, 24.03)	21.83 (19.51, 24.29)	20.66 (17.06, 23.37)	0.91	0.92	0.88
HDL-4 Apo-A1	mg/dL	65.53 (59.38, 72.74)	64.64 (57.7, 72.08)	65.02 (57.17, 73.57)	63.95 (58.76, 69.93)	0.99	0.99	0.98
HDL-1 Apo-A2	mg/dL	1.85 (1.17, 2.54)	1.39 (0.78, 2.09)	1.48 (0.83, 2.27)	1.26 (0.7, 1.82)	0.75	0.80	0.68
HDL-2 Apo-A2	mg/dL	2.51 (2.01, 3.04)	2.17 (1.64, 2.8)	2.29 (1.7, 2.83)	2.05 (1.51, 2.51)	0.86	0.91	0.82
HDL-3 Apo-A2	mg/dL	4.82 (3.93, 5.49)	4.36 (3.78, 5.14)	4.41 (3.84, 5.27)	4.24 (3.52, 4.87)	0.90	0.91	0.88
HDL-4 Apo-A2	mg/dL	15.34 (12.97, 17.42)	15.58 (13.73, 17.45)	15.67 (13.88, 17.54)	15.09 (13.42, 17.35)	1.02	1.02	0.98

Abbreviations: highGS, high Gensini score unstable angina; NCA, normal coronary arteries; lowGS, low Gensini score unstable angina; UA, unstable angina.

Table S3. List of discriminating variables selected as discriminating lipoprotein variables to classify UA, lowGS, highGS and NCA in a pairwise model.

Lipoprotein variables	Unit	NCA vs UA (LP = 10)	NCA vs lowGS (LP = 0)	NCA vs highGS (LP = 32)
TC	mg/dL			
TG	mg/dL			
LDL-C	mg/dL			
HDL-C	mg/dL			selected
Apo-A1	mg/dL			selected
Apo-A2	mg/dL			
Apo-B	mg/dL			
LDL-C/HDL-C	-/-			
Apo-B/Apo-A1	-/-			selected
Total Particle Number	nmol/L			
VLDL Particle Number	nmol/L			selected
IDL Particle Number	nmol/L			
LDL Particle Number	nmol/L			
LDL-1 Particle Number	nmol/L			
LDL-2 Particle Number	nmol/L	selected		
LDL-3 Particle Number	nmol/L			
LDL-4 Particle Number	nmol/L			
LDL-5 Particle Number	nmol/L			
LDL-6 Particle Number	nmol/L			
IDL Triglycerides	mg/dL			
LDL Triglycerides	mg/dL			
HDL Triglycerides	mg/dL			
VLDL Triglycerides	mg/dL			
VLDL Cholesterol	mg/dL			
IDL Cholesterol	mg/dL			
VLDL Free Cholesterol	mg/dL			
IDL Free Cholesterol	mg/dL			
LDL Free Cholesterol	mg/dL			
HDL Free Cholesterol	mg/dL			selected
VLDL Phospholipids	mg/dL			selected
IDL Phospholipids	mg/dL			
LDL Phospholipids	mg/dL			
HDL Phospholipids	mg/dL	selected		selected
HDL Apo-A1	mg/dL			selected
HDL Apo-A2	mg/dL			
VLDL Apo-B	mg/dL			selected
IDL Apo-B	mg/dL			
LDL Apo-B	mg/dL			

VLDL-1 Triglycerides	mg/dL			
VLDL-2 Triglycerides	mg/dL			selected
VLDL-3 Triglycerides	mg/dL			selected
VLDL-4 Triglycerides	mg/dL	selected		selected
VLDL-5 Triglycerides	mg/dL			
VLDL-1 Cholesterol	mg/dL			selected
VLDL-2 Cholesterol	mg/dL			
VLDL-3 Cholesterol	mg/dL			
VLDL-4 Cholesterol	mg/dL			
VLDL-5 Cholesterol	mg/dL			
VLDL-1 Free Cholesterol	mg/dL			
VLDL-2 Free Cholesterol	mg/dL			
VLDL-3 Free Cholesterol	mg/dL			selected
VLDL-4 Free Cholesterol	mg/dL			
VLDL-5 Free Cholesterol	mg/dL			
VLDL-1 Phospholipids	mg/dL			
VLDL-2 Phospholipids	mg/dL			selected
VLDL-3 Phospholipids	mg/dL			selected
VLDL-4 Phospholipids	mg/dL			selected
VLDL-5 Phospholipids	mg/dL			
LDL-1 Triglycerides	mg/dL			
LDL-2 Triglycerides	mg/dL			
LDL-3 Triglycerides	mg/dL			
LDL-4 Triglycerides	mg/dL			
LDL-5 Triglycerides	mg/dL			
LDL-6 Triglycerides	mg/dL			
LDL-1 Cholesterol	mg/dL	selected	selected	
LDL-2 Cholesterol	mg/dL	selected		selected
LDL-3 Cholesterol	mg/dL			selected
LDL-4 Cholesterol	mg/dL			
LDL-5 Cholesterol	mg/dL			
LDL-6 Cholesterol	mg/dL			
LDL-1 Free Cholesterol	mg/dL			
LDL-2 Free Cholesterol	mg/dL	selected		selected
LDL-3 Free Cholesterol	mg/dL			selected
LDL-4 Free Cholesterol	mg/dL			
LDL-5 Free Cholesterol	mg/dL			
LDL-6 Free Cholesterol	mg/dL			
LDL-1 Phospholipids	mg/dL			
LDL-2 Phospholipids	mg/dL	selected		selected
LDL-3 Phospholipids	mg/dL			selected
LDL-4 Phospholipids	mg/dL			
LDL-5 Phospholipids	mg/dL			

LDL-6 Phospholipids	mg/dL			
LDL-1 Apo-B	mg/dL			
LDL-2 Apo-B	mg/dL	selected		
LDL-3 Apo-B	mg/dL			
LDL-4 Apo-B	mg/dL			
LDL-5 Apo-B	mg/dL			
LDL-6 Apo-B	mg/dL			
HDL-1 Triglycerides	mg/dL			
HDL-2 Triglycerides	mg/dL			
HDL-3 Triglycerides	mg/dL			
HDL-4 Triglycerides	mg/dL			
HDL-1 Cholesterol	mg/dL			selected
HDL-2 Cholesterol	mg/dL	selected		selected
HDL-3 Cholesterol	mg/dL			selected
HDL-4 Cholesterol	mg/dL			
HDL-1 Free Cholesterol	mg/dL			selected
HDL-2 Free Cholesterol	mg/dL			selected
HDL-3 Free Cholesterol	mg/dL			
HDL-4 Free Cholesterol	mg/dL			
HDL-1 Phospholipids	mg/dL			selected
HDL-2 Phospholipids	mg/dL			selected
HDL-3 Phospholipids	mg/dL			
HDL-4 Phospholipids	mg/dL			
HDL-1 Apo-A1	mg/dL			selected
HDL-2 Apo-A1	mg/dL			selected
HDL-3 Apo-A1	mg/dL	selected		
HDL-4 Apo-A1	mg/dL			
HDL-1 Apo-A2	mg/dL			
HDL-2 Apo-A2	mg/dL			
HDL-3 Apo-A2	mg/dL			
HDL-4 Apo-A2	mg/dL			

Abbreviations: highGS, high Gensini score unstable angina; NCA, normal coronary arteries; lowGS, low Gensini score unstable angina; UA, unstable angina.

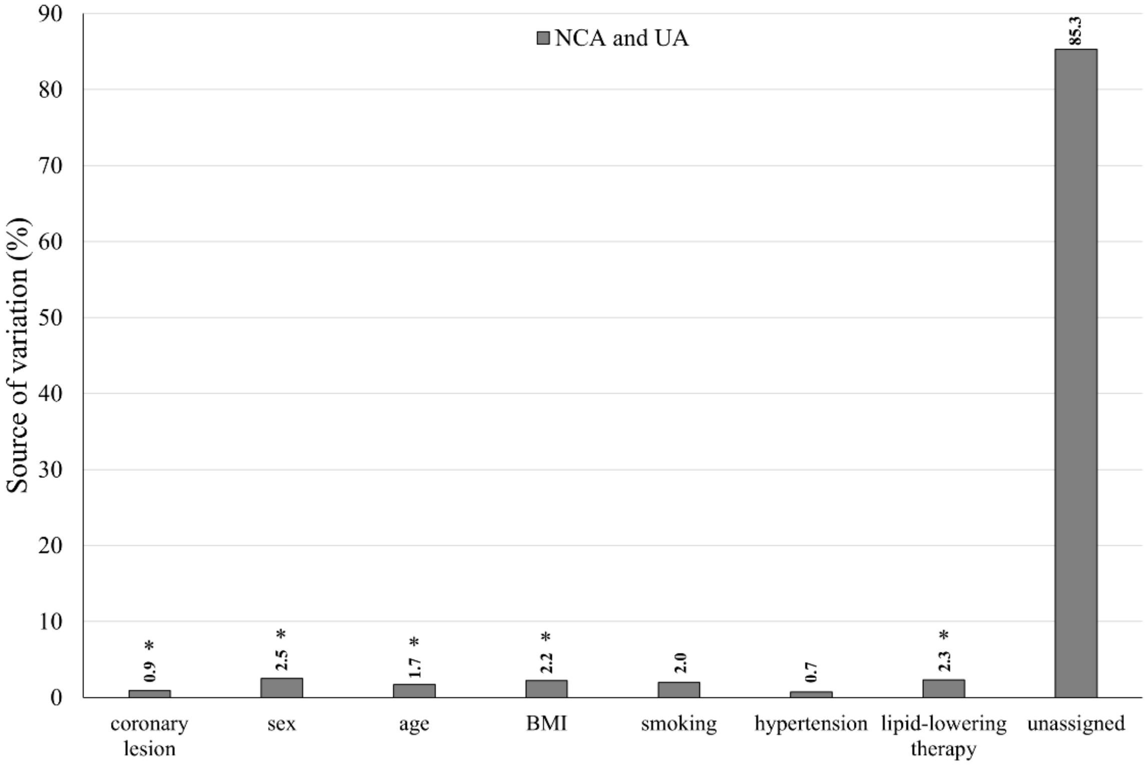

Figure S1. ASCA analysis of the effect of specific covariables on lipoprotein variation of patients with unstable angina (UA) and those with normal coronary arteries (NCA). The relative contribution of specific factors to overall variation was assessed in UA and NCA. Each bar describes how much of the observed variability can be explained by variations in a specific variable. The unassigned group therefore captures both variation resulting from systematic errors and variation resulting from underlying biological states. Abbreviations: ASCA, ANOVA-simultaneous component analysis; NCA, normal coronary artery; UA, unstable angina. *$p<0.05$

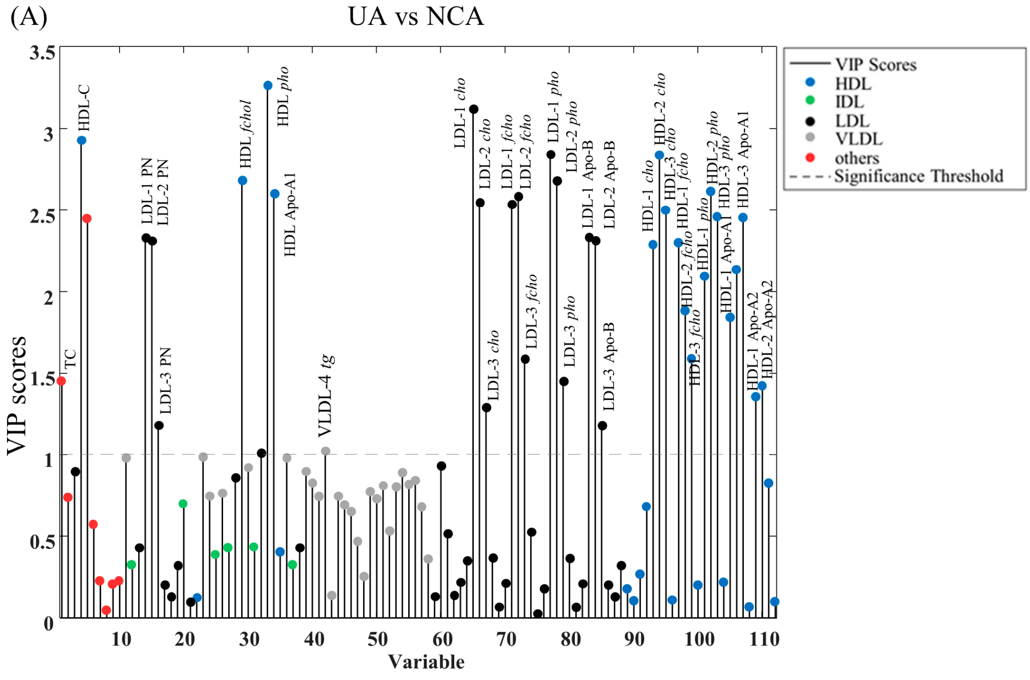

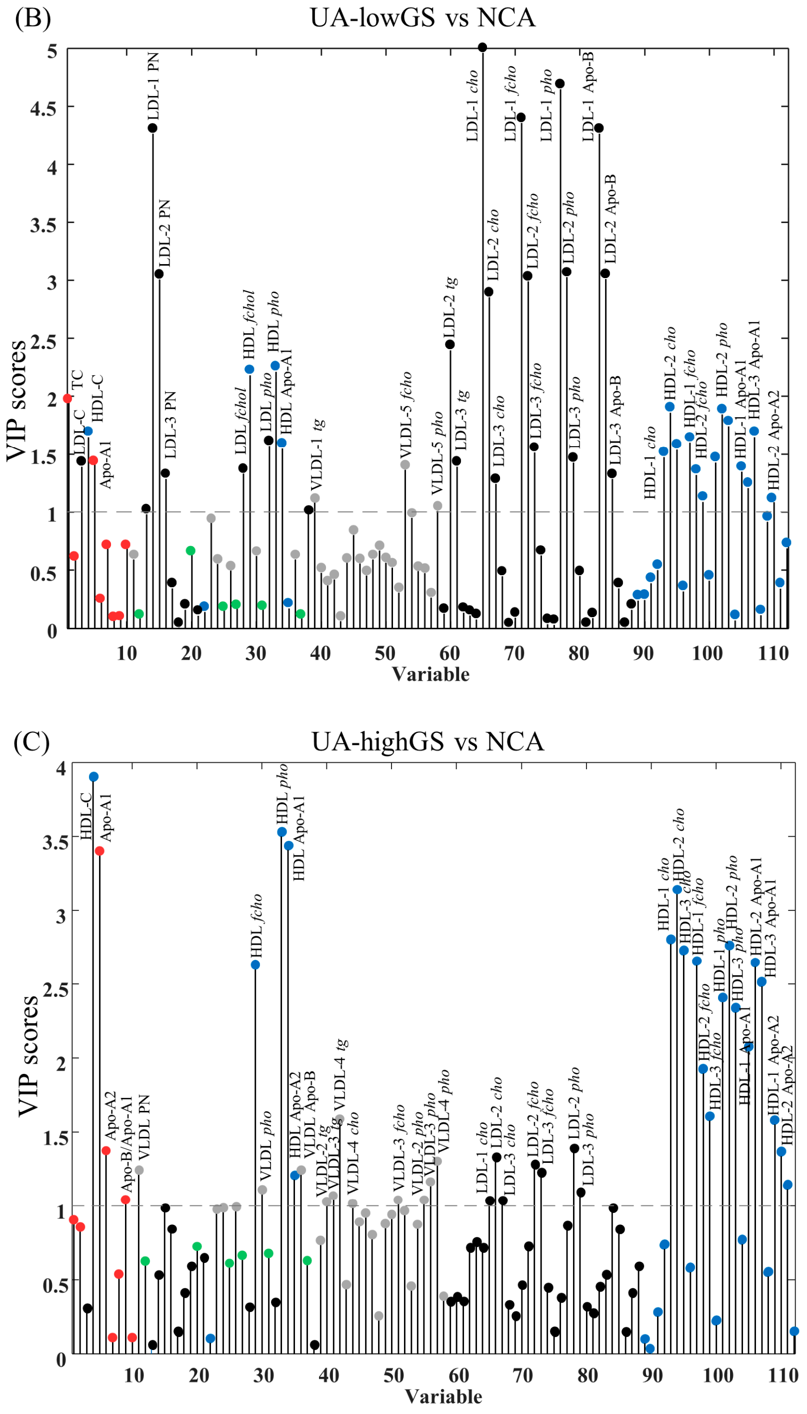

Figure S2. The variable importance in projection (VIP) score plot of 112 lipoprotein variables in partial least squares-discriminant analysis (PLS-DA) of three pairwise comparisons: (A) unstable angina (UA) and patients with normal coronary arteries (NCA), (B) UA patients with low Gensini score (UA-lowGS, GS ≤25.4) and NCA group, (C) UA patients with high Gensini score (UA-highGS, GS ≤25.4) and NCA group. The lipoprotein variables with VIP >1 were considered as most significantly variables to separate two groups. Lipoprotein variables were clustered by lipoprotein particles, which blue represents for HDL, green for IDL, black for LDL, grey for VLDL and red for others. Abbreviations are as in Table S2.

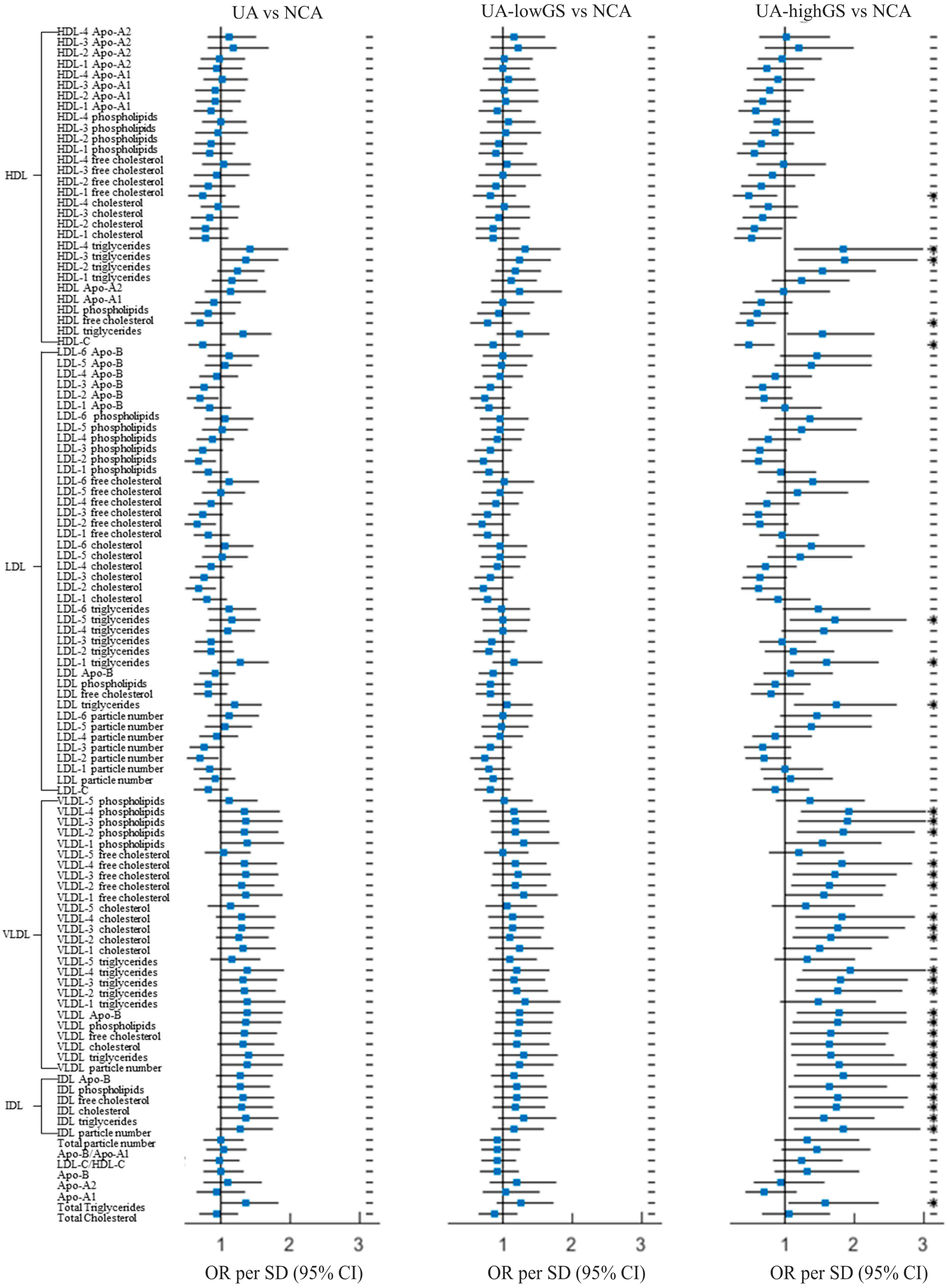

Figure S3. Associations of 112 Lipoprotein Variables with Unstable Angina (UA) in the UA patients without lipid-lowering treatment at baseline (n=223). Data are adjusted odds ratios (OR, blue dots) with 95% confidence intervals (black lines) for per 1-SD (standard deviation) higher levels of lipoprotein variables obtained by logistic regression models adjusting for sex, age, body mass index in the patients without lipid-lowering treatment at baseline (n=223). Significance (Sig.): **FDR-p <0.05, *FDR-p <0.1, -FDR-p >0.1 (p value). Apo-A1, apolipoprotein A1; Apo-A2, apolipoprotein A2; Apo-B, apolipoprotein B; HDL, high-density

lipoprotein; high-GS, unstable angina patients with high Gensini score; IDL, intermediate-density lipoprotein; LDL, low-density lipoprotein; low-GS, unstable angina patients with low Gensini score; VLDL, very low-density lipoprotein.
